# Cauda Canis: Variation of a Tinel's Sign for a Sciatic Nerve Tumor

**DOI:** 10.1155/2020/8822866

**Published:** 2020-12-14

**Authors:** Keith George, Shane Burke, Knarik Arkun, Ron Riesenburger

**Affiliations:** ^1^Department of Neurosurgery, Tufts Medical Center, 800 Washington Street, Boston 02111, MA, USA; ^2^Department of Pathology, Tufts Medical Center, 800 Washington Street, Boston 02111, MA, USA

## Abstract

A patient with a prior history of intradural schwannoma and disc herniation presented with radicular pain after being hit in the thigh by a dog's tail. She was worked up and found to have a tumor of her right sciatic nerve. The tumor was resected and histology was consistent with schwannoma. The dog's tail acted as a Tinel's sign maneuver and led to timely identification of her peripheral nerve tumor. Peripheral nerve schwannomas can present in unusual forms, and Tinel's maneuver may be a useful tool in diagnosis.

## 1. Introduction

Radicular pain in patients undergoing neurosurgical evaluation can be attributed to a variety of causes. A majority of these causes include pathology at the spinal level, principally disc herniation, spinal stenosis, and degenerative changes leading to bone spurs [1]. However, alternative peripheral causes of radicular pain should also be considered part of the full workup. We present an interesting case and the first of its kind, where a woman was found to have a tumor of her sciatic nerve after she was hit in her thigh by a dog's tail (cauda canis translates to dog's tail in Latin) and subsequently developed radicular pain.

## 2. Case Presentation

**Figure 1:**
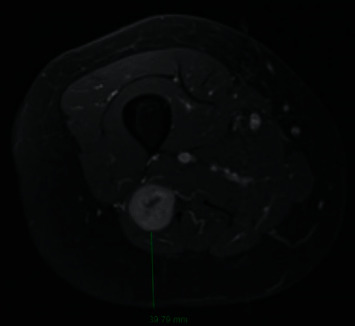
MRI axial showing right sciatic nerve tumor, 4 cm from the skin.

A 57-year-old female, with a prior history of intradural L2 lumbar schwannoma that was resected 7 years ago, presented with right leg pain that radiated into her lateral leg, down to her ankle and big toe. A year prior to her current visit, she was found to have an L4-L5 herniated disc with right-sided radiculopathy and deferred surgical intervention, and improved with conservative measures. After some time, she was hit in the back of her right thigh by the tail of a Labrador dog, and she buckled and noticed severe electric pain shooting down her leg, akin to a Tinel's sign finding. The pain occasionally awakened her and was worse when she sat and improved with activity. She did not endorse a personal history or family history of neurofibromatosis. Physical exam was notable for 4/5 strength in her right anterior tibialis, 4/5 right extensor hallucis longus, decreased sensation to the lateral aspect of the right ankle, and positive Tinel's sign on the posterior thigh. An MRI of the right thigh showed a tumor along the right sciatic nerve, 3 cm in size (Figure 1).

A preoperative ultrasound was obtained to help localize the tumor for surgery. Ultrasound showed the center of the mass 17 cm rostral to the skin crease of the popliteal fossa, with the caudal section of the mass 16 cm from the crease and the rostral portion 18.5 cm from the crease.

**Figure 2:**
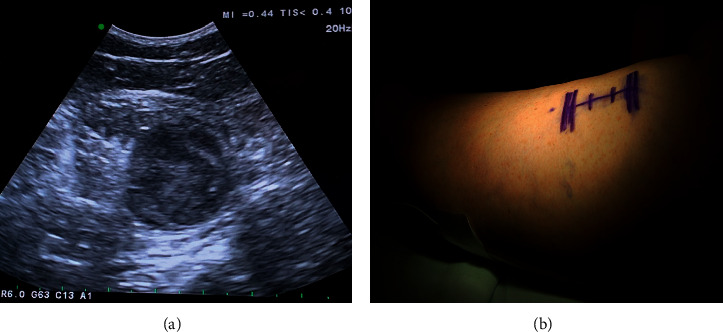
(a) Intraoperative ultrasound localizing tumor. (b) Planned incision along the right posterior thigh.

**Figure 3:**
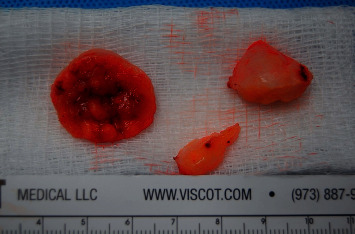
Gross specimen of schwannoma.

**Figure 4:**
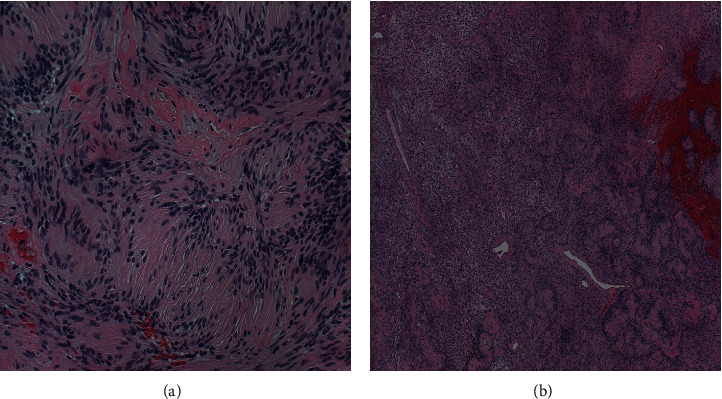
(a) H&E high magnification (X200) of Antoni A area with Verocay bodies formation. (b) H&E low magnification (X40) of biphasic spindle cell lesion with cellular Antoni A areas and numerous Verocay bodies formation on the right, along with many hyalinized vessels and fresh hemorrhage, characteristic of schwannoma. Hypocellular area with looser stroma or Antoni B pattern is on the left. Pigment laden macrophages are present around vessels at the bottom of the image, representing evidence of old microbleed.

**Figure 5:**
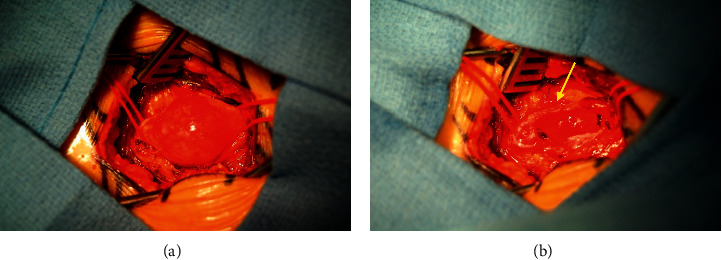
(a) View of the surgical field with tumor along the sciatic nerve. (b) Tumor excised with sciatic nerve left intact (yellow arrow).

During the procedure, the patient was placed prone, and an intraoperative ultrasound confirmed that the tumor was 18 cm from the popliteal fossa (Figure 2). An incision was made along the right posterior thigh, and muscle was dissected until the sciatic nerve tumor was exposed. Intraoperative electromyography (EMG) monitoring was used to delineate tumor versus intact nerve. A section of tumor was sent for pathology, and the tumor was histologically confirmed to be a schwannoma (Figures 3 and 4). Debulking was avascular, and the tumor was excised from the nerve, with minimal fascicle loss (Figure 5). The patient woke without a deficit. The patient recovered well postoperatively with no complications. She was followed up for six weeks after the surgery and was found to have an improvement in symptoms, with good healing of her surgical incision and 5/5 strength bilaterally in her lower extremities.

## 3. Discussion

This is a unique case of an underlying tumor being suspected through a patient's physical contact with a dog's tail (cauda canis sign), and the first of such a case to our knowledge. The dog's tail whipping against the posterior thigh and the resultant electric radiating pain the patient experienced can be related to a positive Tinel's sign, which was reproducible on exam. The radicular pain likely arises from the tumor impinging on the sciatic nerve. Other cases of sciatic nerve schwannomas associated with Tinel's sign have been described in the literature [2–4]. Kralick et al. describe a similar case where a patient shared similar symptoms and had difficulty sitting or lying in bed and also had a positive Tinel's sign on physical exam. Initial radiographic studies could not identify a source for the radicular pain, and as a result of the Tinel's sign, a lower extremity MRI was obtained that captured the tumor, allowing the patient to be adequately treated. In our case, the patient's presentation could have initially been attributed to a recurrence of her prior L4-L5 disc herniation. Were it not for the positive Tinel's sign as a result of the dog's tail, there could have been a delay in identifying the true cause of the patient's symptoms.

Histological analysis showed the tumor to be a schwannoma, with characteristic Antoni A and Antoni B patterns, along with the presence of Verocay bodies. Schwannomas are encapsulated nerve sheath tumors of Schwann cells. They are the most common form of peripheral nerve tumor, accounting for about 35% of all benign peripheral nerve sheath tumors [5–7]. Despite this, involvement of the sciatic nerve is still rare, typically less than 1% of cases [8].

Given her history of a prior schwannoma that was intradural, the patient could have an underlying pathology. A population-based study found that 90% of schwannomas are sporadic, while 3% occurred in patients with neurofibromatosis type 2 (NF2) and 2% in patients with schwannomatosis [9]. NF2 is an autosomal dominant disease that is linked to mutations of the NF2 gene, a tumor suppressor gene, located on chromosome 22. Inactivation of this gene predisposes a patient to developing various tumors in the nervous system [10]. Patients with schwannomatosis are also susceptible to tumor spread along the nervous system, but the genes implicated in pathogenesis are distinct from the NF2 gene [11]. Differentiating between NF2 and schwannomatosis is difficult, as diagnosis is determined by clinical criteria [12]. The patient's diagnosis will depend on gene testing and follow-up MRI. If there is an absence of a vestibular tumor on MRI and no evidence of an NF2 gene mutation, this patient will qualify for a diagnosis of schwannomatosis. She will be followed up by both neurosurgery and neuro-oncology. Family testing will also be recommended.

## Data Availability

Data on the case report are restricted by patient privacy.
